# Is Micellar Catalysis Green Chemistry?

**DOI:** 10.3390/molecules28124809

**Published:** 2023-06-16

**Authors:** Fabrizio Fabris, Markus Illner, Jens-Uwe Repke, Alessandro Scarso, Michael Schwarze

**Affiliations:** 1 Dipartimento di Scienze Molecolari e Nanosistemi, Università Ca’ Foscari Venezia, Via Torino 155, Mestre, 30172 Venezia, Italy; 2 Process Dynamics and Operations Group, Technische Universität Berlin, Straße des 17. Juni 135, Sekr. KWT9, 10623 Berlin, Germany; 3 Department of Chemistry, Technische Universität Berlin, Straße des 17. Juni 124, Sekr. TC-08, 10623 Berlin, Germany

**Keywords:** surfactant, micellar catalysis, green chemistry, water, sustainability

## Abstract

Many years ago, twelve principles were defined for carrying out chemical reactions and processes from a green chemistry perspective. It is everyone’s endeavor to take these points into account as far as possible when developing new processes or improving existing ones. Especially in the field of organic synthesis, a new area of research has thus been established: micellar catalysis. This review article addresses the question of whether micellar catalysis is green chemistry by applying the twelve principles to micellar reaction media. The review shows that many reactions can be transferred from an organic solvent to a micellar medium, but that the surfactant also has a crucial role as a solubilizer. Thus, the reactions can be carried out in a much more environmentally friendly manner and with less risk. Moreover, surfactants are being reformulated in their design, synthesis, and degradation to add extra advantages to micellar catalysis to match all the twelve principles of green chemistry.

## 1. Introduction

**Figure 1:**
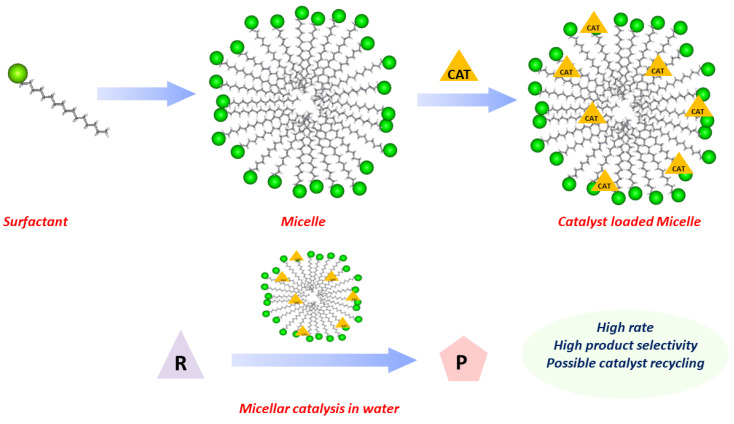
Self-aggregation of surfactants in water driven by the hydrophobic effect provides micellar aggregates that can load a catalyst, acting as nanoreactors for chemical transformations with improved rates, yields, and selectivities by avoiding the extensive use of organic solvents.

“Green chemistry” is a concept that was introduced many years ago, in a period in which the directions for research were focused on achieving new fundamental knowledge and its potential transfer into everyday life. Today, the driving force for research is still the same but current environmental awareness has completely changed the chemical scenario. Environmental protection, sustainability, and a deep understanding of the necessity to make a rapid change in the global mindset about production and consumption have finally achieved consensus, at least in the scientific community and largely also in the overall human community. Nowadays, in developed countries, chemistry is facing a fundamental revolution and each chemical transformation, reagent, or experimental condition is now subject to evaluation for possible environmental impact and overall sustainability [1]. We are rapidly moving towards making all chemistry intrinsically green, hopefully within a few decades, while forcing a re-evaluation of every chemical transformation under this viewpoint [2]. The ACS Green Chemistry Institute [3], REACH (registration, evaluation, authorization and restriction of chemicals) [4], CHEM21 (Chemical Manufacturing Methods for the 21st Century) [5], EPAs [6], and ECHA [7] are playing fundamental roles in this green transition through the education of the chemistry community. The twelve principles of green chemistry [8,9] represent a series of guidelines to move forward in this direction, initially with impact on academia but rapidly also on chemical and all other production companies. When considering chemical transformations, the solvent plays a fundamental role, as it is in most cases the chemical species present in the largest quantity and also exhibits a large potentially negative impact. Because of this, the fifth principle of green chemistry clearly states to avoid, whenever possible, the use of solvents and additives. This for instance spurred the development of mechanochemistry [10], for which a recent review article discusses the several advantages of this synthetic method under the viewpoint of the twelve principles of green chemistry [11]. If a solvent is unavoidable, it should be as innocuous as possible. Solvents are fundamental species for guaranteeing contact and mixing between all reagents and catalysts. Moreover, solvents are not just mediators, in fact they heavily influence chemo-, regio-, and in particular stereo-selectivity [12] in many reactions. Solvent selection is therefore not an easy task and is further aggravated by the extra requirements to improve overall sustainability [13] or avoid toxic solvents [14,15], as clearly agreed by many pharmaceutical companies [16,17,18]. Here, biobased solvents have been proposed to improve defossilization [19]. However, among many new green solvents, water is, and remains [14,20], the solvent par excellence [21,22]. Catalysis in water or on water [23] are known strategies. Nevertheless, a more general and viable approach to cope with the low solubility of organic reagents and metal catalysts in water is to make use of surfactants and the spontaneous formation of micellar aqueous media. The aggregates formed by the hydrophobic effect in water act as nanoreactors (Figure 1) [24,25,26]. This leads to higher local concentrations with rate enhancement and specific solvation effects [27,28], positively affecting product chemo-, regio-, and stereo-selectivity [29].

**Figure 2:**
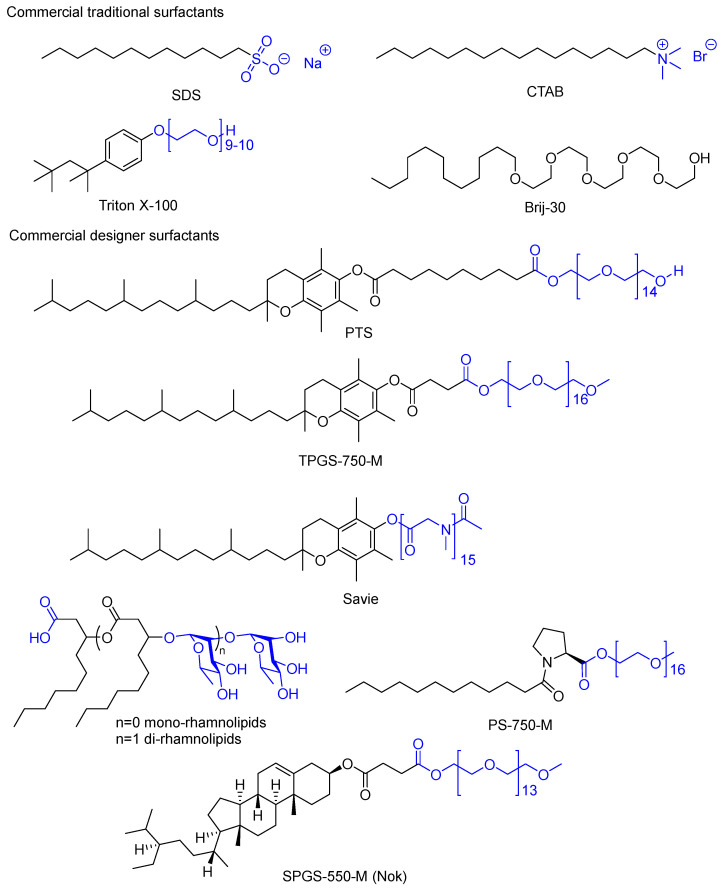
Chemical structures of commercially available traditional surfactants and designer surfactants commonly employed in micellar catalysis.

Figure 2 reports the chemical structures of the most commonly employed commercial surfactants, including those originally developed many years ago for detergency and recently introduced designer surfactants [30] such as TPGS-750-M [31], Nok [32], and PS-750-M [33,34,35,36,37] as typically neutral surfactants specifically designed for catalysis in water under micellar conditions. Micellar media are nowadays a viable alternative to organic solvents for basically every kind of catalytic as well as non-catalytic chemical transformation, as confirmed by a series of review articles published over the years [38,39,40,41,42].

In this critical review, we highlight a series of landmark examples of micellar catalytic systems developed in the period of the last 10 years, which are analyzed in particular under the viewpoint of the twelve principles of green chemistry. In consideration of the impressive burst that the field of micellar catalysis has witnessed in recent years, we believe that this review will encourage the reader to appreciate the full potential of this medium. Based on the proper combination of water and amphiphilic surfactant molecules combined with the ambition for implementation of micellar media, a large push forward to reach the largest possible academic and, more importantly, chemical company audience is attainable.

## 2. Discussion

In the following section, each principle of green chemistry will be rapidly recapitulated, analyzed, and interpreted in the field of micellar catalysis. The viewpoint of the authors is broad, ranging from synthetic organic chemistry to chemical engineering, to catalysis and process development. This permits the authors to provide a balanced overview of the field of micellar catalysis and its green aspects. 

### 2.1. Prevention

**Figure 3:**
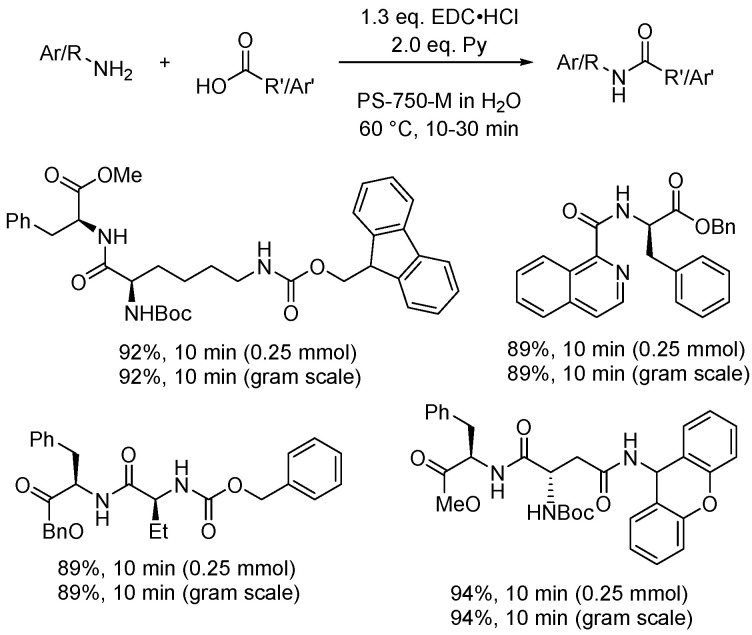
Amide bond formation in water mediated by PS-750-M as a surfactant, promoting the very rapid, gram-scale synthesis of peptides avoiding the use of hydroxybenzotriazole as an additive.

Prevention in the framework of green chemistry means acting to minimize waste formation through a wise selection of reaction and experimental conditions. Solvents are undoubtedly the chemical species most abundant in chemical reactions. Estimates report that about 60% of the mass of chemicals in pharmaceutical chemical industries are solvents. What is highly impactful for the environment and waste production is the fact that most of these solvents are incinerated after use to recover energy, while reuse and downcycling are still limited. Additionally, the incineration of organic solvents leads to CO_2_ production, for which solvents contribute heavily to the amount of waste formed [43] and to a smaller degree to climate change. Quantitative analyses on waste amount formation are commonly analyzed considering the E factor, PMI [44], and other [45,46,47] common green chemistry metrics applied to reactions run in organic solvents. Organic solvent replacement with micellar media frequently allows decreasing such values by even one order of magnitude. This does not mean that organic solvents are never used in micellar catalysis. Minimal amounts of co-solvents are used to (a) improve catalytic performance, (b) improve reactant solubility, or (c) recover products from the micellar media at the end of the reaction. Nevertheless, the amount of organic solvent is greatly decreased when operating in micellar media, thus providing a large advantage in terms of waste prevention. To showcase examples of large waste prevention using micellar catalysis, we selected amide bond formation as a well-established chemical reaction that is commonly carried out using condensing agents, activators operating in common polar aprotic solvents like dimethylformamide, dimethylacetamide, *N*-methyl-pyrrolidone, and dichloromethane. These are solvents of high concern that need to be replaced considering the most recent solvent selection rules. Handa and collaborators have proposed the use of the designer surfactant PS-750-M containing a tertiary amide functional group like most common polar aprotic solvents to carry out amide bonding in water. The reaction was optimized using 1-ethyl-3-(3-dimethylamino)propyl)carbodiimide as a common coupling agent without the use of hydroxybenzotriazole as an additive (Figure 3) [48]. Experimental conditions were optimized leading to a process that led to direct precipitation of the amide product that was subsequently isolated by filtration to completely avoid the use of any amount of organic solvent. 

**Figure 4:**
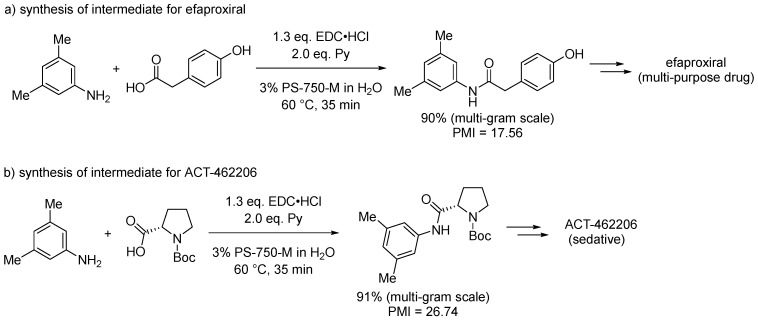
Efficient amide coupling in water with PS-750-M leading to no solvent use and direct isolation of the product by filtration with high overall waste reduction compared to common polar aprotic solvents (**a**) and application of the amide coupling to large-scale synthesis of bioactive compounds (**b**).

The authors further optimized the reaction and applied the methodology to bioactive compounds, synthesized on a multigram scale as intermediates for efaproxiral, a radiation enhancer useful to patients with brain metastases, and ACT-462206 as a potential candidate for the treatment of insomnia (Figure 4) [49]. In both cases, the reaction protocol confirmed high yields, short reaction times, and the absence of any epimerization side reaction.

**Figure 5:**
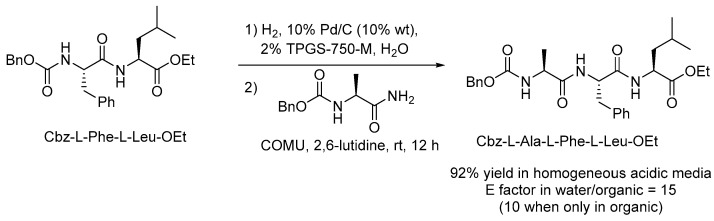
Direct deprotection and amide coupling for peptide synthesis in water under TPGS-750-M micellar medium.

Amide coupling for peptide synthesis consists of sequences of deprotection of the terminal amino group of a growing peptide and the subsequent reaction with an *N*-protected amino acid with a free carboxylic unit. Lipshutz has proposed a tandem one-pot deprotection/coupling reaction for peptide synthesis in water at room temperature mediated by TPGS-750-M (Figure 5) [50]. Specifically, the carboxybenzyl (Cbz) protecting group was removed by hydrogenolysis in a micellar medium with a heterogeneous 10% Pd/C catalyst followed by peptide coupling using 1-cyano-2-ethoxy-2-oxoethylidenaminooxy)dimethylamino-morpholino-carbenium hexafluorophosphate (COMU) as the condensing agent providing a wide range of polypeptides with up to 10 amino acid residues. The new protocol is characterized by the use of very low organic solvent amounts for product isolation, enabling E factors (E factor = kg of waste/kg of desired product) of 15 or 10, if considering or excluding water as waste in the calculation. Such values are up to 100 times lower compared to traditional deprotection and coupling reactions run in organic media.

**Figure 6:**
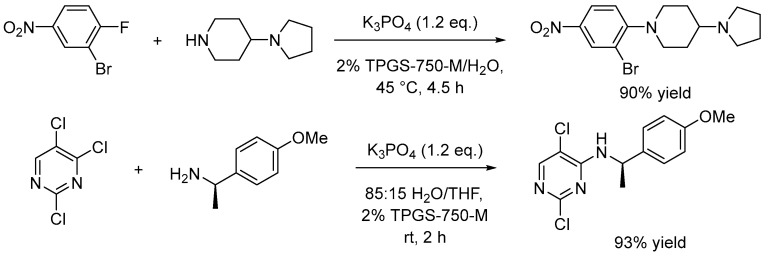
SNAr reaction leading to multigram synthesis of aromatic products containing new C-heteroatom bonds in water.

Another typical chemical reaction run in solvents like DMF and similar polar aprotic solvents is the nucleophilic aromatic substitution (SNAr). This is also one of the most common reactions in the pharmaceutical industry; therefore, waste minimization due to solvent removal is nowadays mandatory. Lipshutz and collaborators reported the ∼10 g scale synthesis of aromatic products via SNAr in water using the well-established designer surfactant TPGS-750-M, applied to two specific reactions, as shown in Figure 6 [51]. The authors optimized several parameters of the reaction and observed that the use of small amounts of co-solvents like THF were crucial to ensure good yields, limited side product formation, and facilitate workup and handling. Finally, ground reagents were observed to be fundamental for drastically decreasing the reaction time, in particular for the second reaction in Figure 6.

**Figure 7:**
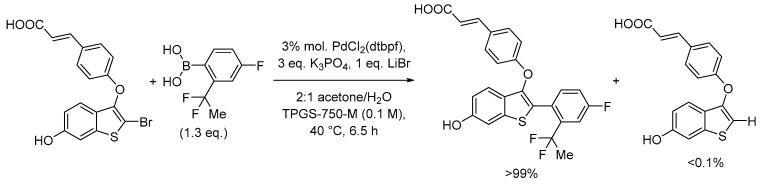
Kg scale Suzuki–Miyaura cross-coupling in TPGS-750-M water micellar media for the synthesis of a drug candidate with a large waste reduction compared to the use of organic solvents.

Micellar catalysis has been demonstrated to be a sufficiently mature methodology for a transition from academic to pharma lab synthesis. Upscaling is a common method to decrease waste formation thanks to increased concentrations and the need to reduce volumes. Suzuki–Miyaura is another important cross-coupling reaction frequently encountered in pharmaceutical synthesis and, recently, a general kilogram-scale protocol has been reported for the synthesis of the drug candidate LSZ102 based on TPGS-750-M in water (Figure 7) [52]. The authors emphasized the positive effect of the use of micellar media also on the reduction of a common de-halogenation by-product, whose formation was reduced to <0.1%. It was estimated that the micellar media approach with respect to the organic medium approach led to a 41% reduction in the PMI index from 116 to 68, which corresponds to an almost equivalent decrease in waste generation. They also evaluated the amount of wastewater coming from the micellar medium with respect to the traditional organic medium and found that they are rather comparable due to the high concentrations of reagents used with micelles and not requiring an intensive aqueous extraction of organic phases. An economic evaluation also underlined the higher costs (about 33%) of the scaled-up reaction in organic media with respect to micellar media based on raw materials and processing costs.

**Figure 8:**
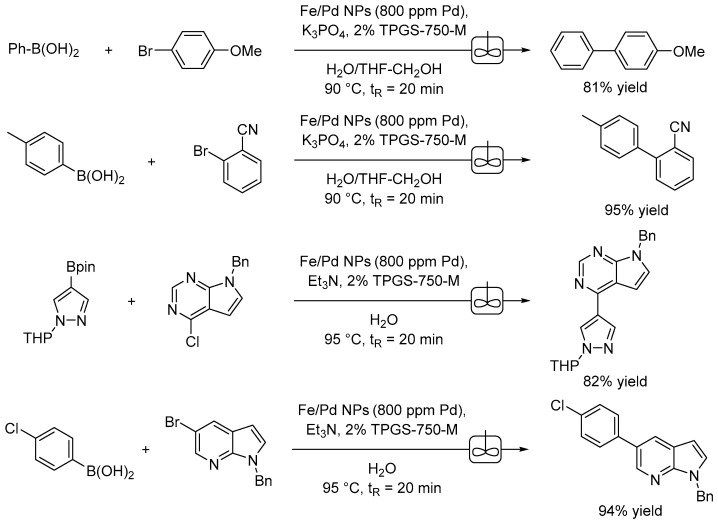
Continuous flow Suzuki–Miyaura cross-coupling reactions in water with TPGS-750-M enabling the synthesis of different intermediates for bioactive compounds.

When aiming to scale up a reaction by one or two orders of magnitude, the use of flow chemistry represents a viable solution for optimizing and running the reaction for a longer time and with positive effects on waste reduction. Micellar catalysis is compatible with flow chemistry as described by Lipshutz and collaborators in a paper in which they used Fe/ppm Pd nanoparticles with TPGS-750-M in water for a series of Suzuki–Miyaura couplings (Figure 8) [53]. Specifically, the authors used a continuous stirred-tank reactor (CSTR) employing only 800 ppm of Pd (i.e., 0.08 mol%) in the form of Fe/ppm Pd.

**Figure 9:**
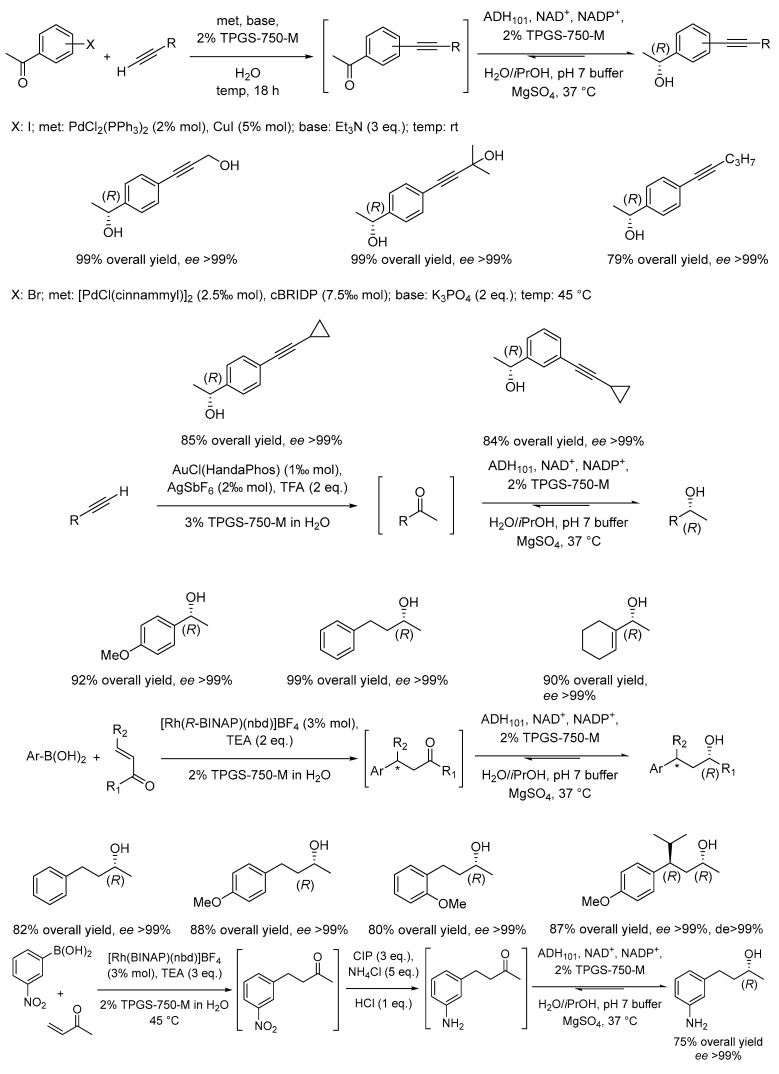

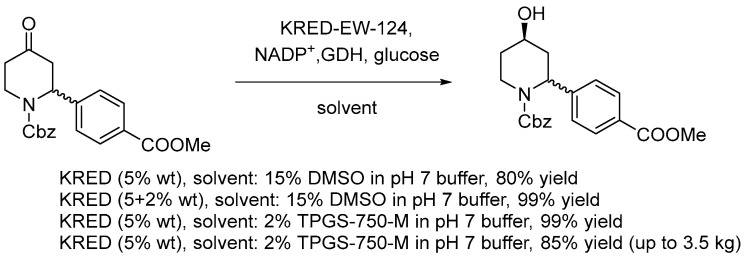
Selected examples of telescoped metal-catalyzed and enzyme-catalyzed reactions enabled by the use of TPGS-750-M micellar medium (ADH alcohol dehydrogenase, KRED ketoreductase).

Concerning large waste reductions, it is additionally important to consider that dimethyl sulfoxide (DMSO) or dimethylformamide (DMF) are frequently added to overcome poor solubility of organic substrates in water with enzymes. This poses a significant impairment of the green aspects of bio-catalysis in water. Moreover, sequences of metal- and enzyme-catalyzed steps are frequently encountered in organic synthesis, each with specific solvent requirements and hence leading to high waste generation. This challenge was addressed by the Lipshutz group by proposing the use of TPGS-750-M-based micellar media to link several metal- and enzyme-catalyzed reaction steps in a one-pot approach [54]. Specifically, a series of Pd, Cu, Rh, Fe, and Au catalyzed reactions could be performed in sequence with stereoselective reduction steps mediated by alcohol dehydrogenase leading to highly stereo-enriched products of greater complexities (Figure 9). To prove the versatility of the micellar medium as an alternative to the commonly employed water/DMSO mixtures in enzymatic catalysis, the authors performed a >3 Kg sized enzymatic reduction of a cyclohexanone derivative with 85% isolated yield of the corresponding enantioenriched 4-piperidinol (Figure 9).

**Figure 10:**
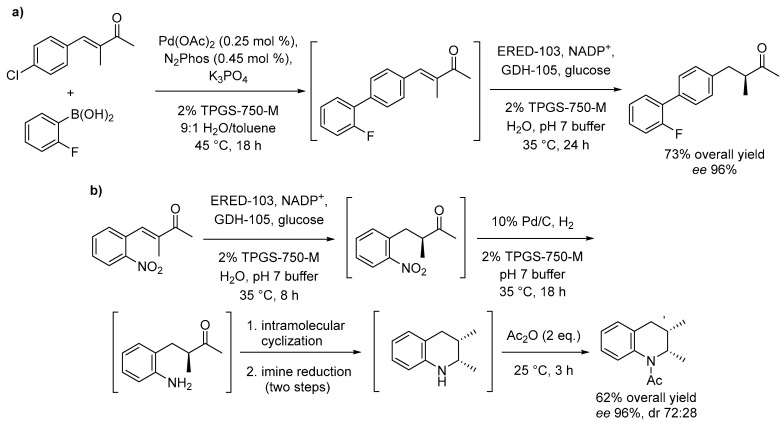
One-pot tandem Pd-catalyzed cross-coupling followed by enzymatic stereoselective reduction leading to the final enantioenriched ketone product in water in the presence of TPGS-750-M (**a**) and one-pot four step combining two chemo- and two bio-catalytic reactions all mediated by the same micellar medium based on TPGS-750-M (**b**).

Lipshutz and collaborators demonstrated that the use of micellar media also enables the direct tandem sequence between two biocatalysts as well as between a metal catalyzed reaction and a subsequent enzyme mediated step. Specifically, the authors reported a one-pot chemo- and bio-catalytic reaction resulting from the combination of a cross-coupling reaction mediated by Pd catalysts with a reductase (ERED)-catalyzed stereoselective unsaturated carbonyl compounds reduction (Figure 10) [55]. Other one-pot combinations of up to four chemo- and bio-catalytic steps were achieved as described in Figure 10b. 

### 2.2. Atom Economy

**Figure 11:**
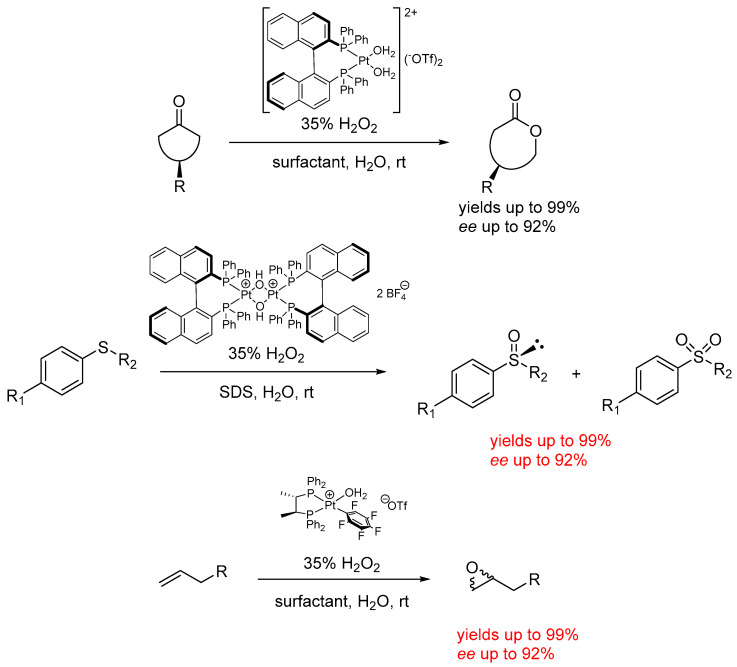
Asymmetric Baeyer–Villiger, sulfoxidation, and epoxidation reaction with atom-economic hydrogen peroxide as terminal oxidant under aqueous micellar conditions.

The optimization of the incorporation of most of the atoms of the reagents into the products is an important green chemistry principle that is well exemplified in oxidation reactions. Typically, many atoms from the oxidant are lost when aiming for the incorporation of one oxygen atom in the substrate. Among many oxidants, hydrogen peroxide is surpassed only by molecular oxygen in terms of atom efficiency for oxidation reactions. Micellar catalysis is inherently suitable for the use of hydrogen peroxide in oxidation reactions, which leads to water as a benign by-product and already-present solvent. Moreover, hydrogen peroxide is usually deployed as a solution in water at different concentrations, and thus in a form which is completely compatible with micellar conditions. Several examples of oxidation reactions under micellar conditions were reported about ten years ago by the group of Strukul and Scarso, focusing also on the effects of the confinement provided by the micelles on the stereoselectivity for asymmetric oxidation reactions (Figure 11). It was observed that every substrate and catalyst combination required specific optimization of the nature of the surfactant and the concentration of all species to ensure a high product yield with high enantioselectivity.

Using chiral Pt(II)-diphosphine catalysts, the authors observed that the asymmetric Baeyer–Villiger oxidation of cyclic meso-cyclobutanones in micellar media led to high yields and an *ee* (enantiomeric excess) of up to 56%. For meso-cyclohexanones, the enantioselectivity increased up to 92%, in both cases with better results with respect to what is observed in organic media [56]. Similarly, the asymmetric sulfoxidation of aryl alkyl thioethers with hydrogen peroxide, mediated by the chiral platinum diphosphine complex [(BINAP)Pt(*μ*-OH)]_2_(BF_4_)_2_, led to enhanced chemoselectivity as well as stereoselectivity, operating under micellar media with respect to dichloromethane as the organic solvent (16% *ee* in dichloromethane and 40% *ee* in water/SDS) for the oxidation of thioanisole [57]. As far as the asymmetric epoxidation of the terminal alkenes is concerned, the chiral Pt(II) complex {[(*S*,*S*)Chiraphos]Pt(C_6_F_5_)(H_2_O)}(OTf) with hydrogen peroxide under a micellar medium allowed comparable conversions with respect to dichloroethane as an organic solvent in the epoxidation, but with a remarkable increase in enantioselectivity from 58% *ee* to 82% *ee*, with also the possible recycling of the system over three consecutive runs [58]. 

**Figure 12:**
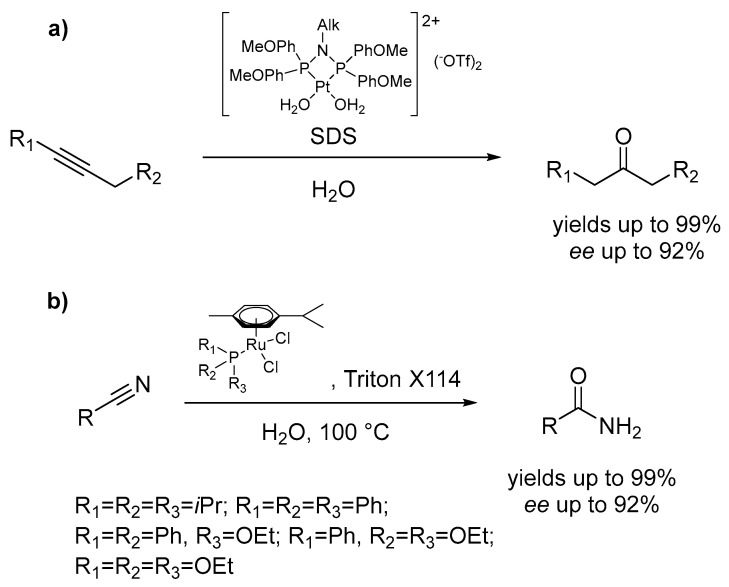
Hydration of terminal alkynes with Pt(II) catalyst with water and SDS (**a**) and hydration of nitriles with Ru(II) catalyst in water and Triton-X114 (**b**) under micellar conditions.

Hydration reactions, in which water is a reagent, are naturally predestined for being carried out under micellar conditions to avoid the use of organic co-solvents for improving the solubility of the substrate. Examples have been reported for terminal alkyne hydration forming respective carbonyl products corresponding to Markovnikov hydration mediated by platinum catalysts that could be recycled up to four times without loss of catalytic activity (Figure 12a) [59]. In the latter case, it is worth mentioning that the careful catalyst conditioning within the solution showed the specific role played by the micellar aqueous medium favoring the formation of catalytically active Pt(II) species bearing σ vinyl residues, while in water–organic media the reaction stops at the π-coordinated alkyne species. Similarly, also the nitrile hydration of the corresponding amide products mediated by Ru(II) catalysts has been developed in micellar media (Figure 12b) [60] in which the surfactant-containing medium enabled catalytic activities comparable to those achieved with water soluble Ru(II) catalysts.

**Figure 13:**
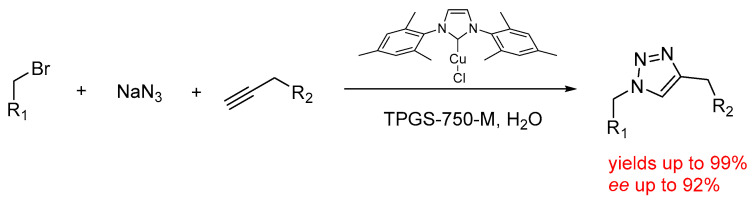
Three-component triazole synthesis in water under micellar conditions avoiding isolation of noxious alkyl azide products.

It is also worth mentioning that other intrinsically atom efficient reactions, e.g., cycloadditions, have been extensively optimized under micellar conditions. For instance, the three-component reaction between alkyne, alkyl halide, and sodium azide has also been achieved avoiding the isolation of potentially harmful alkyl azide compounds. The reaction was optimized using a proper active Cu(I) catalyst (1 mol%) in water at room temperature with the aid of TPGS-750-M as a surfactant. The reaction was extended to a large range of alkynes and alkyl halides obtaining the corresponding 20 different 1,2,3-triazoles with 51 to >98% yields in water (Figure 13) [61], in some cases also on a 2 mmol scale within a few hours.

### 2.3. Less Hazardous Chemical Syntheses

**Figure 14:**
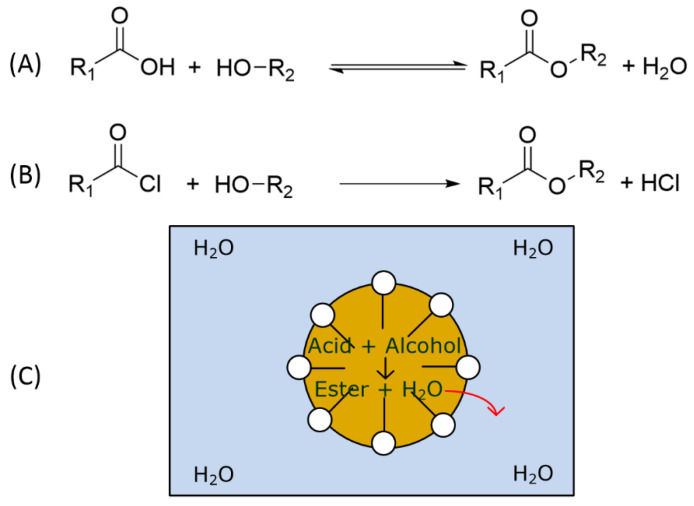
Esterification using an acid (**A**), an acid chloride (**B**), and under micellar conditions (**C**). The red arrow indicates the release of produced water to the continuous aqueous phase.

This principle focuses on alternative reaction pathways that avoid hazardous reactants or auxiliaries to synthesize the desired product. As micellar solutions only act as green solvents, they have no direct influence on the selection of the reactants for the chosen reaction. Only through surfactant screening, an optimal reaction performance is guaranteed. However, there are examples where the micellar solution allows the use of safer chemicals for the synthesis. Figure 14 shows the scheme of an esterification reaction performed under different conditions. 

An esterification reaction is the reaction between an acid and an alcohol to produce an ester and water as the by-product. The water can also hydrolyze the ester in a backward reaction to produce the acid and the alcohol so that esterification is basically an equilibrium reaction. To avoid the backward reaction, a hazardous acid can be used to turn the reaction into an irreversible reaction. Specifically, acid chloride can be used, which leads to the formation of HCl as an additional harmful by-product. If a micellar reaction medium is used, the esterification proceeds within the micelles, while the produced water will leave the hydrophobic interior towards the hydrophilic continuous phase. The surfactant and reaction conditions can be selected in such a way that after the reaction the hydroxyl ions, which are responsible for the hydrolysis, have no contact with the ester formed. Hence, the backward reaction is inhibited. There are many articles about hydrolysis reactions in micellar media and the impact of the surfactant on the reaction rate. Usually, ester hydrolysis is accelerated using cationic surfactants able to bind the hydroxide ions [62] so that an anionic surfactant should be beneficial for the esterification reaction. Abe et al. showed that in anionic micellar solutions under neutral and alkaline conditions, the hydrolysis of the ester is much slower than under acidic conditions [63]. Hastings et al. studied the hydrolysis of p-nitrophenyl esters showing that nonionic surfactants like TPGS-750-M are also able to suppress the hydrolysis, if the ester is encapsulated in the micellar core. When the ester forms mixed micelles with the surfactant, the ester can get into contact with the hydroxide ions, resulting in an increased hydrolysis rate [64]. Cationic surfactants with different chain lengths were investigated by Schmidt et al. for the hydrolysis of methyl decanoate showing that the reaction rate increased for shorter surfactants possibly forming smaller micelles [65]. 

### 2.4. Designing Safer Chemicals

The fourth principle of green chemistry states that chemical products should be designed to preserve or increase efficacy of function but at the same time limiting as much as possible toxicity and side effects for the environment. This implies the substitution of harmful chemicals with other substances which ensure comparable performance but are characterized by a much better safety and toxicological profile. Because of this, the substitution of applied organic solvents with water is with no doubt a great improvement concerning chemical reactions. In micellar catalysis, the nature of the surfactant becomes crucial in order to assess the real toxicity of the medium. It is also important to consider that surfactant loading is commonly in the range of 1–5% *w*/*w* when considering the aqueous solution; therefore, the mass amount used is almost two orders of magnitude smaller with respect to the corresponding amount of organic solvent in traditional reactions. Surfactants are commonly employed in everyday life for a multitude of applications, whereas the largest amounts are used in detergency. Among the many different classes of surfactants, anionic species like sodium alkyl benzene sulfonate and sodium lauryl ether sulfonate cover almost 50% of the market of commercial use with overall almost 20 Mmt (millions of tons) of production per year. Neutral species like alkyl ethoxylate ethers represent about 35% market coverage with the residual 15% consisting of alkylammonium species and zwitterionic compounds for special applications. In particular, commercially available anionic and neutral surfactants have been largely employed in the early years of micellar catalysis development also because of their SDS classification, which does not evidence high risk or particular toxicity. The advent of designer surfactants [66] as specialized amphiphilic neutral compounds designed for catalysis in water has enriched the portfolio of surfactants. Many of these are composed of bio-based apolar organic units like sitosterol, fatty acids, or tocopherol connected with spacers of different natures to polyoxyethylene units as the hydrophilic portion. In consideration of their important applications, some of the most useful designer surfactants are commercially available, such as TPGS-750-M, Nok, and PS-750-M. Nevertheless, their production of small volumes does not require registration, for instance in Europe, with the European Chemical Agency (ECHA), nor are they regulated by the Environmental Protection Agency (EPA) of the USA. Moreover, no data are available on their acute and chronic toxicity as well as proper classification and handling instructions. Certainly, the expansion of their future potential to real production technologies for instance in pharmaceutical industries will spur the full determination of their toxicological profiles. Nevertheless, it is advisable to deploy these new surfactants for micellar catalysis. In particular, biosurfactants typically comprising apolar lipidic units connected to carbohydrate units like sophorolipids and rhamnolipids are still not used for this purpose. Nevertheless, their future investigation is highly advisable. Specifically, the advantages of biosurfactants are related to their high biodegradability and low toxicity. These positive properties are promoting scientific studies on a wide range of industrial applications [67]. A possible limitation of their use in micellar catalysis is related to their variable chemical compositions and, in some cases, their production routes via fermentation leading directly to aqueous solutions containing complex mixtures of compounds. It will be important to investigate their compatibility, for instance, with metal catalysis in water to avoid catalyst deactivation. Overall, the use of micellar media for chemical reactions is completely in agreement with the fourth principle of green chemistry and, together with the fifth principle related to solvent replacement, confirms the great advantages possible by replacing solvents with micellar media. 

### 2.5. Safer Solvents and Auxiliaries

For a very long time, solvents were selected primarily on the basis that all substances could dissolve and react in them to avoid interfaces and thus mass transfer limitations. Since a large number of chemical reactions take place with hydrophobic reactants, nonpolar solvents are state of the art. However, improved solubility often also increases the hazard potential, as many solvents are highly flammable (e.g., cyclohexane), toxic (e.g., dimethyl formamide), or carcinogenic (e.g., benzene). With the introduction of the principles of green chemistry, the term “green solvent” was also coined. Up to now, there is no definition for “green solvents”, but, in a figurative sense, they are solvents that have advantages over classic organic solvents regarding their impact on human health and the environment. To identify green solvents at an early stage, Capello et al. used the EHS (E: Environment, H: Health, and S: Safety) assessment method to rank standard organic and new solvents [68]. The method uses nine effect categories for a solvent including flammability, risk of explosion, decomposition, and toxicity to rank solvents according to an EHS value between zero and nine. A higher EHS value indicates a higher risk associated with the solvent. Table 1 shows the EHS values of selected solvents used in chemical synthesis. The EHS method can also be used to evaluate the risk of solvent mixtures. With respect to the selected nine effect categories, water has an EHS value of zero and is the best green solvent. 

**Figure 15:**
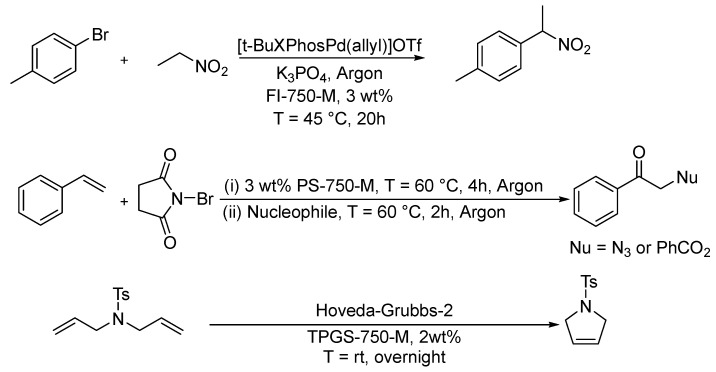
Examples for replacement of classical organic solvents by micellar solutions.

The biggest problem associated with water is the poor solubility of hydrophobic substances. To solve this problem, surfactants are suitable solubilizers. By using micellar solutions, hydrophobic substances can be dissolved and thus reactions can be easily carried out in water, as already shown. The solvent system thus consists predominantly of water and a final EHS evaluation is carried out based on the surfactant. Although there is a wide range of surfactants available, an optimal choice must be made based on the reaction performance (surfactant screening) and in accordance with the principles of green chemistry. This leads to the question, which surfactants “are green”? The fourth principle is closely related to this one and already addresses surfactant properties. Also here, the trend is certainly toward surfactants that can be produced from renewable raw materials, e.g., sugar-based surfactants. By using micellar reaction media, many chemical reactions can now be carried out much more safely. First and foremost, the risk of fire and explosion is eliminated, since water is used as a non-flammable solvent. Caution is then only required if other auxiliary substances are used that can react exothermically with water. Furthermore, many important reactions have been carried out using surfactants to replace harmful solvents to obtain the best catalytic performance. Some examples are shown in Figure 15.

Brals et al. studied the palladium-catalyzed arylation of nitroalkanes [33]. They replaced the toxic organic solvent 1,4-dioxane, commonly used for this type of reaction, with a micellar reaction medium made of the designer surfactant FI-750-M. The micelles have sizes larger than 300 nm providing an ideal reaction environment for the hydrophobic reactants. Using [*t*-BuXPhosPd(allyl)]Otf as the catalyst, a yield of >90% was achieved within 20 h reaction time, whereby the catalyst concentration was in the range of 1–5 mol%. The catalyst could be recycled several times. A calculated E factor of about five shows the greenness of this process. Harmful dipolar-aprotic solvents such as DMF, DMAc, NMP, or 1,4-dioxane are quite often used in organic synthesis. Hazra et al. studied the oxyfunctionalization of styrene in micellar solutions of the designer surfactant PS-750-M [69]. The surfactant was designed to structurally mimic the named solvents and, depending on the applied nucleophile (Nu), azidoketones (Nu = N_3_) or acyloxyketones (Nu = PhCO_2_) were obtained on a gram scale with yields in the range of 60 to 80%. This investigation shows that even challenging compounds that have a tendency for polymerization (styrene) or decomposition (azide), which hence require dipolar-aprotic solvents, could be used safely in micellar solutions. Organic solvents such as toluene, benzene, 1,2-dichloroethane, and dichloromethane (DCM) are typical solvents for Ru-catalyzed ring closing metathesis [70]. In the past, it was unthinkable that metathesis catalysts could be used outside organic solvents without facing deactivation of the catalyst. With the help of micellar solutions, such reactions can also be carried out in an aqueous environment, thus replacing hazardous solvents. Hastings et al. studied chemoenzymatic reactions in micellar solutions [71]. The main idea was to couple a well-studied chemical reaction with a subsequent enzymatic reaction. They show that micellar solutions could be a good environment for coupled reactions. Some of the shown examples also included a ring-closing metathesis (RCM) reaction, e.g., the coupling of *N*,*N*-diallyl-4-methylbenzenesulfonamide to 1-tosyl-2,5-dihydro-1H-pyrrole using a ruthenium Hoveyda–Grubbs second generation catalyst. Under mild reaction conditions, they obtained 98% conversion. Evidently, the designer surfactants have a high solubilization capacity due to the huge size of the micelles and can support many types of reactions. Furthermore, the micelles have a shielding effect and protect the solubilized reactants as well as catalysts from having too much interaction with water. 

### 2.6. Design for Energy Efficiency

Design for energy efficiency aims at reducing energy requirements for the production of a chemical component. Therein, the total energy demand is summarized and related to the amount of substance produced. This entails reaction conditions resulting from the deployed reaction media and the reaction pathway. Alongside the energy input for maintaining reaction temperatures, the pressure is to be considered, as compressor operation costs and energy demand are considerably high at elevated pressures. Furthermore, the resulting exothermic behavior of novel synthesis routes might be favorable when higher reaction temperatures are to be maintained. In addition to that, the energy input required for the extraction of the product from the reaction mixture has to be considered. Especially the latter aspect is one of the key features of micellar reaction media. Exemplarily, the phase behavior of a ternary system of oil, water, and an amphiphile is considered. According to Kahlweit et al. [72], a ternary phase separation state exists for which a middle emulsion phase coexists with an upper oily and a lower aqueous excess phase. Therein, excess phases are characterized by amphiphile concentrations at the critical micelle concentration, which is considerably low with the proper choice of surfactants. For 0–40 °C, critical micelle concentrations of 10^−5^–10^−4^ mol L^−1^ are found for water [73]. Hence, the separation of, e.g., nonpolar substances at high purities is possible by simple means of gravity-driven phase separation. This alone poses an outstanding potential for energy efficiency, since the large amounts of (thermal) energy required for standard distillation sequences can be largely avoided. With a suitable choice of the amphiphile, matching reaction and separation conditions is also possible. Pogrzeba et al. investigated this for aliphatic nonionic surfactants of the Marlipal^®^ type [74]. By adjusting the chain length and the degree of ethoxylation, the surfactant can be chosen to meet the desired reaction conditions (e.g., emulsification of nonpolar reactants and aqueous catalyst solution) and the phase behavior. Hence, it is possible to perform reaction and separation in a similar temperature range and thus avoid cooling and heating cycles for batch or even continuous operation with recycling. This has been already demonstrated for the hydroformylation of 1-dodecene on a mini-plant scale using acetylacetonato(dicarbonyl)rhodium(I) as a catalyst immobilized in an aqueous phase via the ligand SulfoXantPhos [75] and Marlipal^®^ 24/70 as a surfactant [76]. Here, the reaction step is operated at 95 °C and 15 bar, while the phase separation is achieved in a subsequent settler unit, operated at 74–98 °C depending on current reaction yield and microemulsion configuration. The same principle has been successfully demonstrated for the reductive amination of undecanal [77] and the hydroaminomethylation of decene [78].

Regarding the reaction temperature, it is well known that reaction rates increase with temperature and, often, higher temperatures are required for organic synthesis reactions to take place. However, the reaction rate of chemical reactions without the problem of substrate inhibition also increases when the concentration is increased at the same temperature. In micellar catalysis, the hydrophobic reactants are solubilized inside the micelles leading to locally higher substrate concentration. The micelles act as small nanoreactors, in which the stoichiometric or catalytic reaction takes place with high substrate concentrations. Many organic reactions can thus be carried out at room temperature or slightly above as shown in the previous sections due to this micellar effect. Although, a higher substrate concentration is considered as the main effect for the rate enhancement in micellar catalysis in the literature, a recently published simulation-based contribution showed another explanation. Martin P. Andersson investigated the Pd(0)-catalyzed Suzuki–Miyaura coupling with DFT calculations and indicated a loss in entropy (ΔS), which leads to an increase in the prefactor of the kinetic constant, as the main reason for the better performance of organic reactions under micellar reaction conditions [79]. His approach might be used as a tool to study catalytic reactions in micellar environments to select the best micellar-based medium for the reaction.

Since chemical reactions under micellar conditions can also be carried out with less waste and thus a better E factor (Section 2.1), significant energy savings are attainable, since less waste has to be treated. This is obvious for multistep reactions that are transferred from classical organic synthesis routes to micellar media. In the classical route, the reaction product would be isolated and purified before being used in a subsequent reaction step resulting in a huge amount of produced waste. Micellar solutions allow for continuing with the reaction medium without isolation of the product. Here, Handa et al. showed examples for Fe-catalyzed Sonogashira coupling reactions. After a first Sonogashira coupling reaction, the produced alkyne was used for a second Sonogashira coupling (two steps) or for follow-up reactions (5 steps). All reactions could be performed in a one-pot approach in a micellar solution using the designer surfactant TMGS-750-M without product isolation and after completing the reaction sequence, an overall yield of about 80% was obtained [80]. The success of multistep reactions in micellar media is based on the different locations of hydrophilic and hydrophobic reactants. It also allows bridging the gap between bio- and chemo-catalysis as shown by Cortes-Clerget et al. After a Pd-catalyzed Sonogashira coupling, the carbonyl group of the obtained product was reduced using a bio-catalyst in the same reaction medium. For the whole sequence, both high yield and enantiomeric excess were obtained [54]. 

Energy can also be saved by reducing the time of the reaction under otherwise identical reaction conditions. Micellar catalysis has its focus in the field of organic synthesis and the solubilization of hydrophobic reactants in aqueous environments by the micelles. One aspect that has not been studied extensively so far is the influence of surfactants on heterogeneous catalyzed reactions. Here, the solubilization of the hydrophobic reactants, but also the transport of the reactants to the active sites of the usually porous catalyst, must be ensured. Due to the porous structure of the catalyst, mass transfer limitations due to pore diffusion often occur, resulting in poorer reaction performance. However, surfactants tend to adsorb at surfaces and are thus able to form a surfactant layer in the pores of porous materials [81]. Shinde and Bhagwhat showed this for a Pd/C-catalyzed Sonogashira coupling. However, the selection of the surfactant remains crucial, as several of the investigated surfactants are able to solubilize the reactants in water but only CTAB enabled suitable reaction rates [82]. 

Significant energy savings can additionally be achieved by using smaller amounts of organic solvents for product extraction. Another approach with lower energy input can be carried out following the catalyst separation concepts discussed in Section 2.9. Removing homogeneous catalysts from organic solvents can be done by solvent resistant nanofiltration using higher pressures (>1 MPa) and therefore higher energy to obtain a reasonable flux. In contrast, homogeneous catalysts embedded in micelles can be separated by ultrafiltration at much lower pressures (0.1–0.3 MPa) [83]. 

### 2.7. Use of Renewable Feedstocks

**Figure 16:**
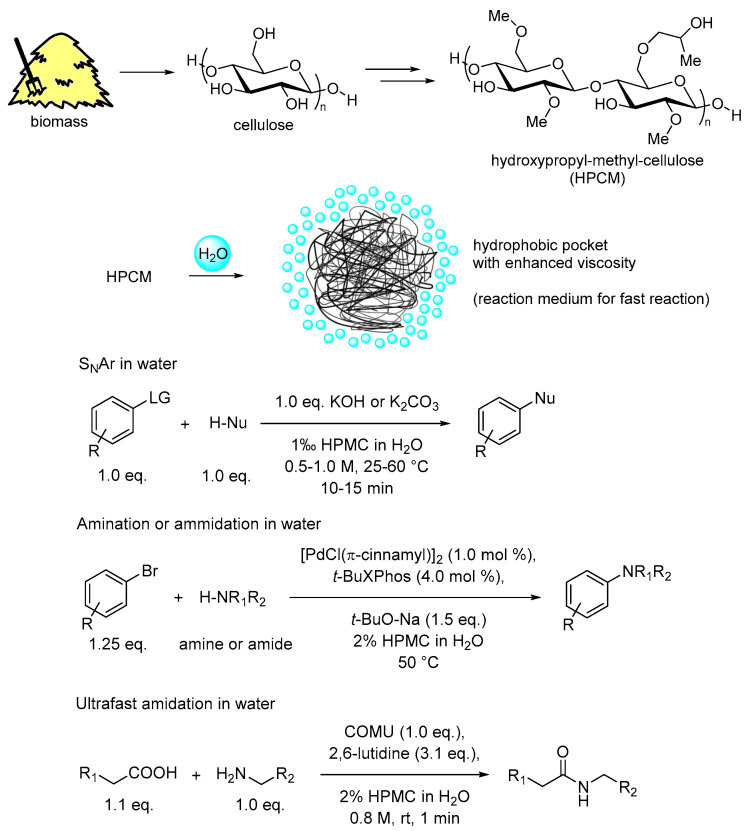
Molecular structure of HPMC and selected examples of extremely rapid reaction in water using this technology.

This principle focuses on favoring as much as possible the usage of renewable feedstocks like bio-based compounds over fossil-based chemicals. The chemical industry’s production of surfactants is almost exclusively derived from petrochemical compounds. Therefore, classical anionic or neutral surfactants do not match this requirement. Emerging designer surfactants represent a substantial change in this regard since all new proposed amphiphilic compounds are composed, at least for the hydrophobic part, of bio-based units. In Figure 2, the most common designer surfactants are displayed, in which tocopherol, belonging to the vitamin E group for TPGS-750-M, sitosterol for Nok, and a common fatty acid for PS-750-M are naturally occurring compounds. In contrast, the hydrophilic parts of these neutral surfactants based on poly oxyethylene units stem from non-renewable sources. They are typically obtained from ethylene through epoxidation to ethylene oxide and subsequent polymerization. To confer water solubility while avoiding the presence of charges, it is envisioned to develop new surfactants, in which for instance carbohydrate-based polar units could be employed. This way, the overall renewable character of designer surfactants could be improved. A complementary approach has recently been proposed making use of the commercially available hydroxypropyl methyl cellulose [84] (HMPC, Figure 16). It can be considered as an inexpensive, non-toxic, and biodegradable polymer in which most of the free hydroxyl units are capped with less polar methyl and hydroxypropyl units. This leads to polymeric compounds with amphiphilic nature that self-organize in water at mild heating to 45–60 °C. Here, hydrophobic pockets able to improve organic substrate solubilization in water are formed. Their application for catalysis in water has led to extremely promising results [85]. This includes, for instance, favoring the Pd-catalyzed amination and amidation of aryl halides, the amide coupling with condensing agents [86], the nucleophilic aromatic substitution, the C−N bond formation via visible-light-mediated photoredox catalysis, and the classical Suzuki–Miyaura cross-coupling, for all of which very short reaction times, mostly within minutes, are attainable. Nevertheless, several disadvantages exist for this methodology. For example, product isolation is difficult and requires sodium sulphate addition. Furthermore, gelation needs to be avoided, requiring precise control of the experimental conditions. Moreover, the polymeric nature of the species, the possible different level of alkylation, and the grade of HPMC are critical factors for the success of this aqueous technology. While the HPMC technology is not as simple and straightforward as traditional micellar catalysis, it represents a viable approach that frequently could be a solution for specific synthetic issues in which micellar catalysis fails. 

Very recently, research groups have started to investigate the application of biosurfactants to classical micellar catalysis reactions like Pd-catalyzed cross-couplings. Novák and collaborators demonstrated that rhamnolipids produced by the microorganism Pseudomonas aeruginosa and characterized by one or two rhamnose hydrophilic carbohydrate units connected to one or two hydrophobic fatty acid chains favor the micellar Suzuki- and Sonogashira-couplings on fluoroalkyl vinyl iodide substrates as well as similar steps in the synthesis of more complex API intermediates [87]. The great advantages of biosurfactants are their high biodegradability and their biological production from renewable feedstocks; both fundamental advantages will further promote the use of biosurfactants in micellar catalysis.

### 2.8. Reduce Derivatives

**Figure 17:**
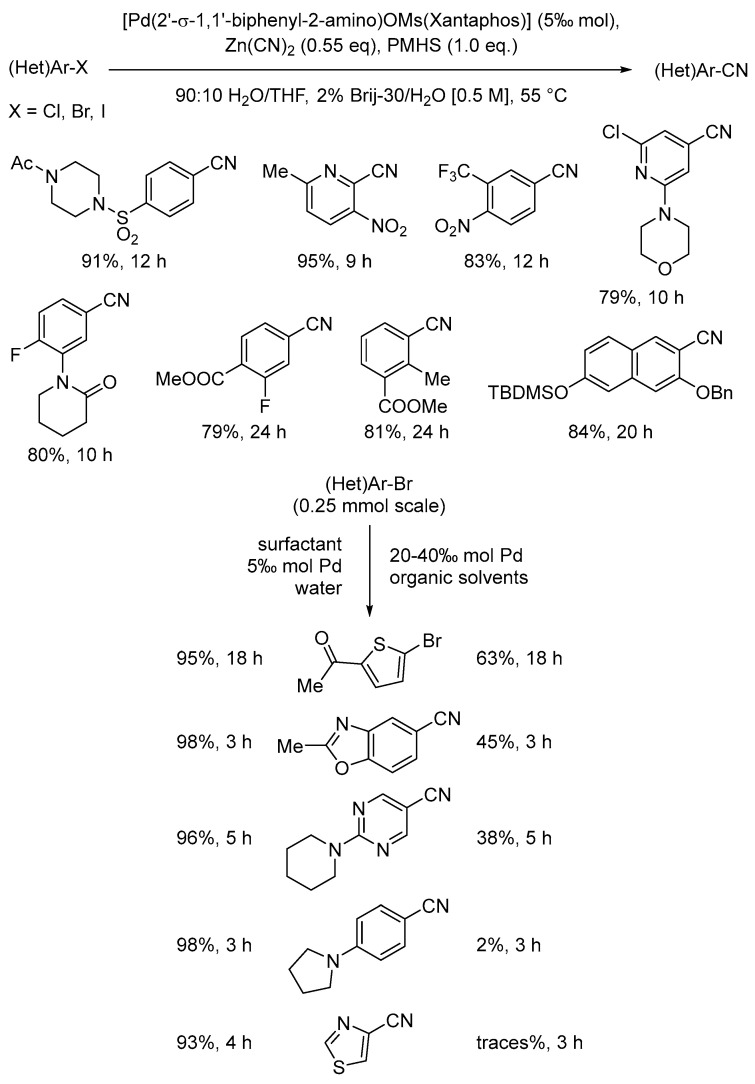
Late-stage cyanation of complex aryl and hetero aryl compounds in water mediated by the neutral surfactant Brij-30.

This specific green chemistry principle focuses on the limitation of the use of protecting groups and any other chemical derivatization that could be avoided through the implementation of new chemical strategies and technologies. In the recent literature concerning micellar catalysis, the Pd-catalyzed late-stage cyanations of aryl and hetero aryl compounds using zinc cyanide in water in the presence of Brij-30 as a neutral surfactant represent an important example of step minimization (Figure 17) [88]. In fact, the installation of nitrile moieties in organic compounds is frequently carried out by de-hydration of the corresponding primary amides, while direct cyanation methods are still scarce and poorly efficient in terms of the high catalyst loading requested. It is also important to mention that this catalytic method in micellar media is compatible with present ketones, esters, as well as protecting groups like triisopropylsilyl (TIPS), *t*-butyldimethylsilyl (TBDMS), benzyl, acetyl, and butyloxycarbonyl (Boc), all well-tolerated by these mild reaction conditions thus avoiding deprotection/reprotection steps, making this reaction highly desirable for applications in the pharmaceutical industry.

**Figure 18:**
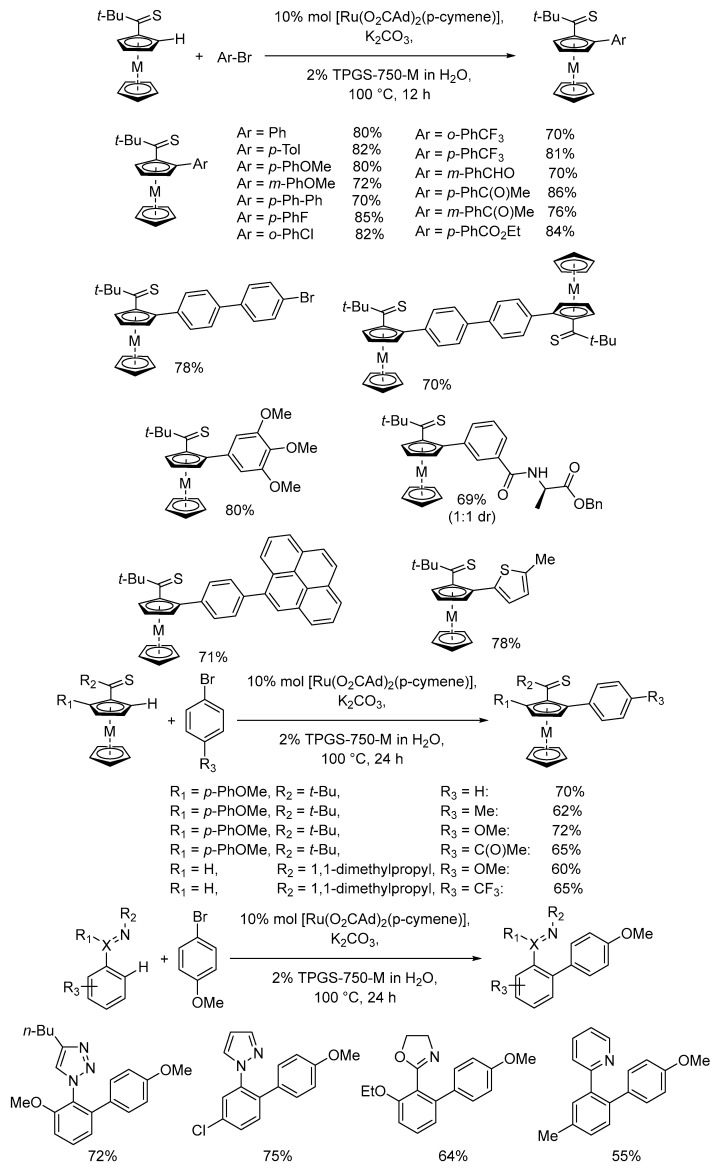
Direct C-H arylation of ferrocenyl compounds mediated by Ru catalyst in water with TPGS-750-M.

The selective direct C–H activation and functionalization of aromatic or aliphatic compounds is a hot topic due to the possibility of installing specific functional groups or creating direct bonds without the need for pre-functionalized coupling partners, which are obtained through several synthetic steps. Micellar catalysis provides important support to this specific kind of transition metal catalyzed reactions [89]. One example is the direct C-H arylation of ferrocenyl compounds endowed with a weakly coordinating thioketone unit by Ru catalyst in water in the presence of TPGS-750-M as a surfactant (Figure 18) [90].

The reaction was highly chemoselective with a very wide range of aryl bromides, in which the thioketone functional group was crucial for the weak coordination with the metal center and the concomitant C–H activation in the ortho position. The robust synthetic protocol also demonstrated the recycling of the micellar medium up to four times. Moreover, a remarkable improvement in waste minimization was calculated. In moving from toluene to the micellar medium, the E factor could be decreased from 14.7 to 5.6.

**Figure 19:**
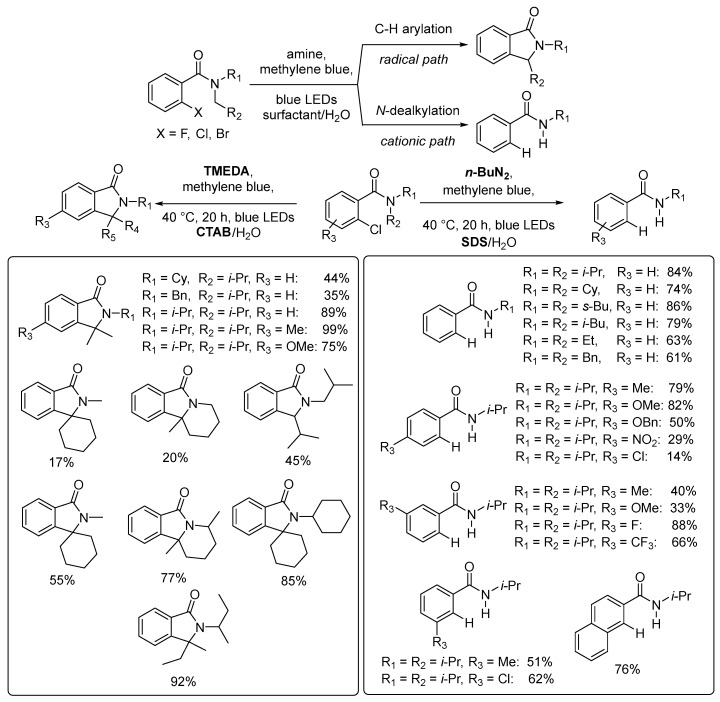
Photocatalytic chemo-divergent functionalization of 2-chloro-benzamide in water with amines leading to isoindolinones or secondary de-halogenated amides by changing experimental conditions, in particular moving from cationic to anionic micellar media.

Micellar catalysis can provide unique mechanistic pathways for specific conditions. Specific chemoselectivities are highly sought after in synthetic organic chemistry because they enable shortcut synthetic pathways replacing traditional methods involving more steps. The development of new chemoselectivities is fundamental to widen the toolbox of organic synthesis. An example of how micellar catalysis can provide new chemoselective methods was reported recently by channeling the photocatalytic reaction of 2-chloro-benzamide into two chemo-divergent reactions: the C–H arylation through intermediate *N*-acyliminium radicals mechanism leading to isoindolinones or the *N*-dealkylation through the intermediate *N*-acyliminium cation forming the corresponding secondary amides (Figure 19) [91].

The catalytic system operates under mild conditions with methylene blue as a photocatalyst and blue LEDs as the light source. The factor determining the reactivity of substrates and product selectivity was the ionic nature of the surfactant. Here, CTAB and TMEDA as base drive the isoindolinones synthesis, while SDS and n-butylamine steer the reaction towards secondary amides with concomitant de-halogenation. The reaction operates under very mild experimental conditions with methylene blue as a photocatalyst. Proper selection of the amine drives the different chemoselectivities of the reaction, further emphasizing the complex nature and the potential of micelle nanoreactors in catalysis.

### 2.9. Catalysis

**Figure 20:**
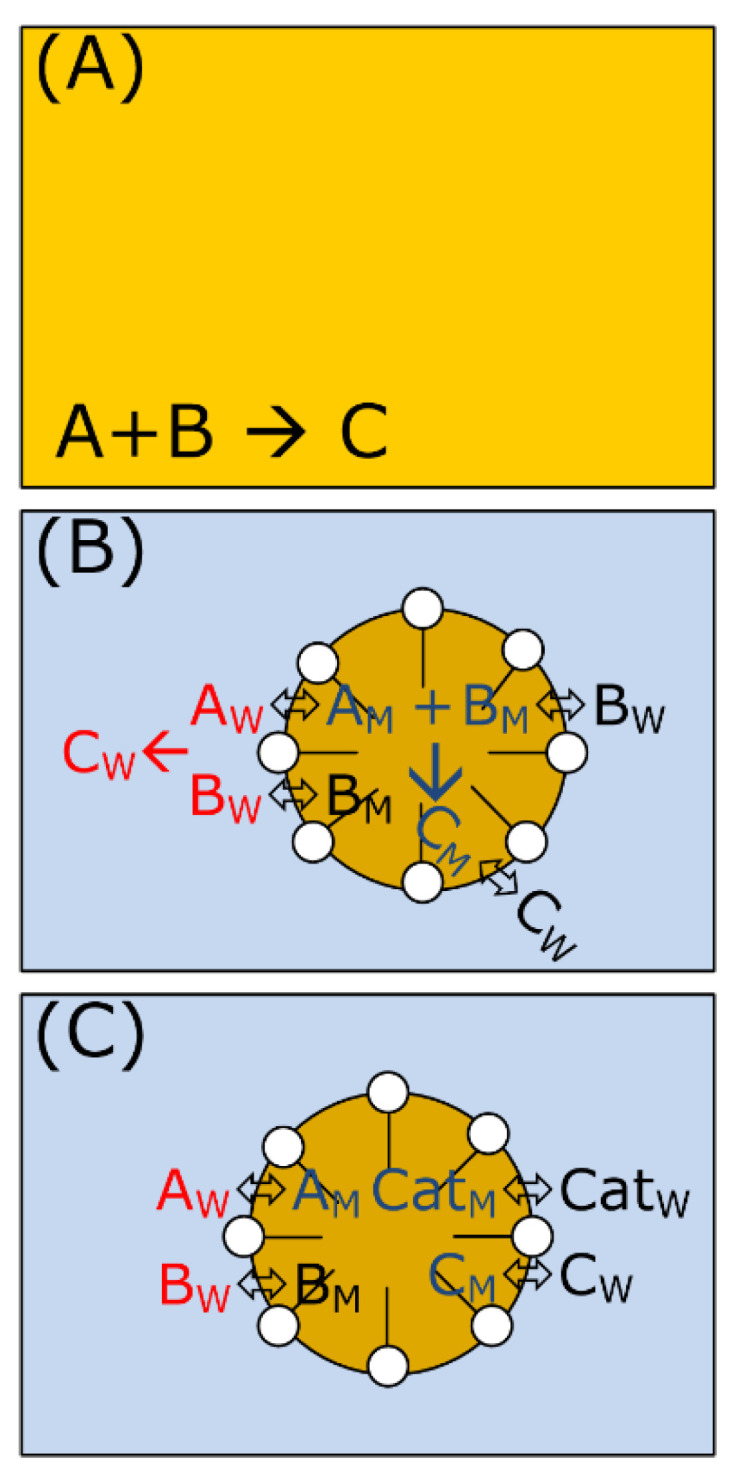
Stoichiometric reaction in organic solvent (**A**), stoichiometric reaction in micellar solution (**B**), and catalyzed reaction in micellar solution (**C**).

Many stoichiometric and catalyzed reactions are carried out in typical organic solvents to ensure an equally good solubility and contact of all reactants (Figure 20A). 

With respect to green chemistry, catalyzed reactions are favored. But the governing question is how to apply this principle to micellar solutions. It is observed that many stoichiometric reactions are accelerated in micellar solutions for which the term “Micellar Catalysis” has been introduced. Some examples are dye fading [92,93] or ester hydrolysis [65,94]. However, surfactant micelles are not catalysts and are subject to a catalytic cycle. Moreover, this effect is attributed to the partitioning of reactants within a microscopic two-phase system (Figure 20B) enabling locally higher reactant concentration in the micellar pseudo-phase. There are two main options for how the micelles can interact with the substrate: (a) hydrophobic interactions and (b) electrostatic interactions. Especially the latter is exploited for reactions involving ions, e.g., hydroxyl ions in hydrolysis reactions, whereby micelles of cationic surfactants (e.g., CTAB) increase the reaction rate as they bind OH^−^ ions close to the reactant. Micelles of anionic surfactants (e.g., SDS) show the opposite effect. For many cases of stoichiometric reactions where the surfactant leads to a rate enhancement, the rate depends on the surfactant concentration and shows a maximum. When the selected surfactant concentration is too high, the reactants are diluted by a larger number of available micelles. This indicates that the local concentration is mainly important for rate enhancement in micellar media [95,96]. To apply the concept of catalysis to micellar solutions, a catalyst has to be added to the system, and it has to be evaluated whether the catalytic reaction benefits from the micellar reaction medium. As shown in the previous sections, many homogeneously catalyzed reactions involving metal complexes can be performed in micellar solution. Surfactant screening is often mandatory to obtain the best catalytic performance. When a catalyst complex is used in a reaction, it will also distribute between the micelles and the surrounding aqueous phase (Figure 20C). Finally, there are different possibilities for the distribution of educts, product(s), and catalyst, which will have an impact on the reaction rate as well as the options for separation afterward. Most of the publications regarding organic transformations in micellar reaction media deal with hydrophobic reactants and a hydrophobic catalyst complex. In this case, all reactants are localized within the micelles, and the reaction is only transferred from a macroscopic hydrophobic reaction medium (organic solvent) to a microscopic hydrophobic environment (micelles). The situation is almost the same (worse separation of catalyst and product), with the advantage of using large amounts of water and a non-hazardous reaction medium. The catalyst is proven to be stable in repetitive batch runs, but for product separation small amounts of an organic solvent are required. 

**Figure 21:**
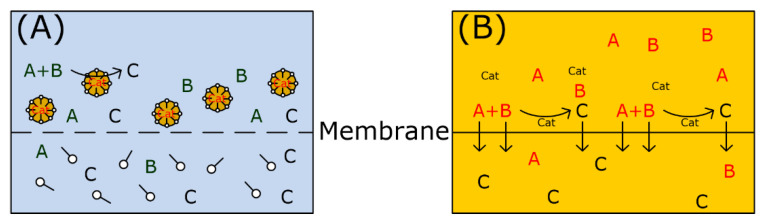
Concept of MEUF (**A**) (with favored partitioning of reactants) and SRNF (**B**) for catalyst and product separation.

Besides their solubilization ability for hydrophobic reactants in water, the advantage of micellar solutions is that surfactants have very different properties, which can be used directly for catalyst and product separation, if there is a favorable distribution of both [97]. This means that the catalyst and the product are in different phases, as it is usually known from two-phase catalysis. The aggregation behavior of surfactants above the critical micelle concentration is the basis for micellar catalysis. Hence, a certain amount of surfactant is required to obtain beneficial features. The micelles are regarded as nanoreactors for the chemical reaction, but they also trap the homogeneous catalyst complex. This offers the possibility to use micellar enhanced ultrafiltration (MEUF, Figure 21A), a concept well-known from water treatment [98], to recycle the catalyst as an alternative method to solvent-resistant nanofiltration (SRNF, Figure 21B). 

Even though the micelles are not rigid spheres, the exchange processes of the monomer units are very fast, so the micelles can be regarded as nanometer-sized agglomerates that can be separated with membranes, if a suitable pore size is chosen. If the catalyst is solubilized by the micelles, it can be recycled, while hydrophilic products can pass the membrane. Due to the size of the aggregates, porous membranes can be used, resulting in acceptable flows even at low transmembrane pressures. This is a distinct advantage over SRNF, where a dense solvent-resistant membrane and significantly higher pressures are required. The application of MEUF is yet challenging and surfactant selection is crucial as it has a dual role. It must support both catalysis and separation. There are only few papers published on MEUF for the recycling of a homogeneous catalyst complex, but, in all cases, catalyst recovery was in the order of 90% and higher [83,99,100]. In addition to the possibility of forming aggregates, micellar solutions are characterized by a complex temperature dependence. Micellar media formed with nonionic surfactants show a phase change from one- to two-phase, when a critical temperature is exceeded. This temperature decreases, if the hydrophobic character increases, usually expressed by a lower hydrophilic–lipophilic-balance (HLB) value. Depending on the density of the surfactant, it forms the lower or upper phase after separation. Dissolved reactants with a high affinity for the surfactant, e.g., a homogeneous catalyst complex, can be separated with the surfactant-rich phase after the temperature has been increased. 

**Figure 22:**
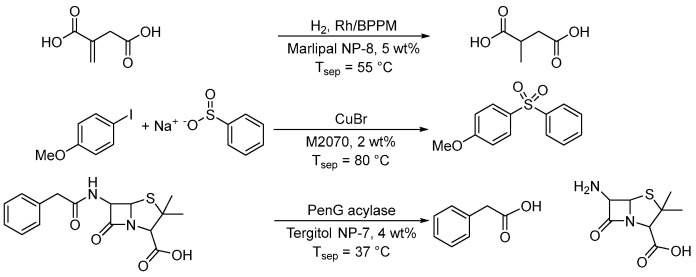
Examples of combined micellar catalysis and extraction.

Figure 22 shows examples for catalyst–product separation after temperature-induced phase separation. Schmidt et al. investigated the Rh/BPPM-catalyzed hydrogenation of itaconic acid to methylsuccinic acid in a micellar solution made of the nonionic surfactant Marlophen NP-8 and showed that after phase separation at 55 °C more than 95% of the hydrophobic catalyst complex is in the surfactant-rich phase [101]. As both itaconic acid and methylsuccinic acid are very hydrophilic, they will accumulate in the water-rich phase. In the case of hydrophobic reactants, a hydrophilic catalyst complex has to be applied. Water-soluble catalyst complexes can be obtained by using water-soluble ligands. The best-known example is the Rh/TPPTS complex from the Ruhrchemie/Rhône-Poulenc process [102]. Another example for temperature-induced phase separation was shown by Ge et al. investigating a copper-catalyzed Ullmann C-S coupling reaction between 1-iodo-4-methoxybenzene and sodium benzenesulfinate to 1-methoxy-4-(phenylsulfonyl)benzene [103]. Using CuBr as the catalyst, a high yield of about 90% was obtained with micellar catalysis. The product was concentrated in the surfactant-rich phase after phase separation at 80 °C and precipitated by cryogenic centrifugation. The authors mentioned that the copper catalyst was complexed by a chelating effect through the amine group of the surfactant. However, there is no analysis of the two phases with respect to copper. When copper is bound to the micelles, it can be assumed that copper is recycled with the surfactant-rich phase after product precipitation. The product extraction efficiency was about 93%. Since the product tends to be hydrophobic, increasing the surfactant concentration is also beneficial in terms of the accumulation of the product in the surfactant-rich phase. For better phase separation after the coupling reaction, 1 wt% NaCl and 10 wt% M2070 were added to the reaction mixture. Even if nonionic surfactants can be separated by raising the temperature, the volume of the surfactant-rich phase is very small. However, in case of small reaction volumes this can make efficient phase separation problematic. This is why a further increase in the surfactant concentration is advantageous for the separation. Ritter and Smirnova studied the bio-catalytic hydrolysis of penicillin G to 6-aminopenicillanic acid and phenylacetic acid in a Tergitol NP-7 based micellar solution [104]. The enzyme was recovered from the water-rich phase after phase separation at 37 °C. Both products show very different polarities and phenylacetic acid is obtained from the surfactant-rich phase while 6-aminopenicillanic acid accumulates in the water-rich phase. The selected model system was used to demonstrate that bio-catalytic reactions in surfactant systems can also be carried out very well in terms of technical implementation and that both batch and continuous operation are possible [105]. There is a huge potential in this combination as enzymes prefer the aqueous environment and will accumulate in the water-rich phase, whereas most of the reactants are hydrophobic and will be located in the surfactant-rich phase after the separation. Furthermore, hydrophobic reactants will be in the micelles with higher concentration during the bio-catalytic reaction leading to an acceleration of the reaction rate in water. Micellar solutions are of interest as green solvents in bio-catalysis as shown by Sheldon et al. [106] and Gröger et al. [107]. 

The best-known property of a surfactant is its ability to produce foam. When a gas stream is dispersed in a surfactant solution, foam bubbles are formed as the surfactant molecules adsorb at the interface between the liquid and the gas. This surfactant property is used in froth flotation to separate pollutants [108,109] or particles [110,111]. Froth flotation is normally carried out at surfactant concentrations close to the CMC in order to discharge a larger proportion of the solute with froth. Flotation also involves the same interactions as in a micellar solution. Thus, charged molecules, e.g., metal ions, can be separated with the foam of an ionic surfactant with opposite charge. Theoretically, it is possible to separate charged catalyst complexes with foam. But so far there is no published work on this topic, although from a technical point of view, flotation would be rather easy to perform.

**Figure 23:**
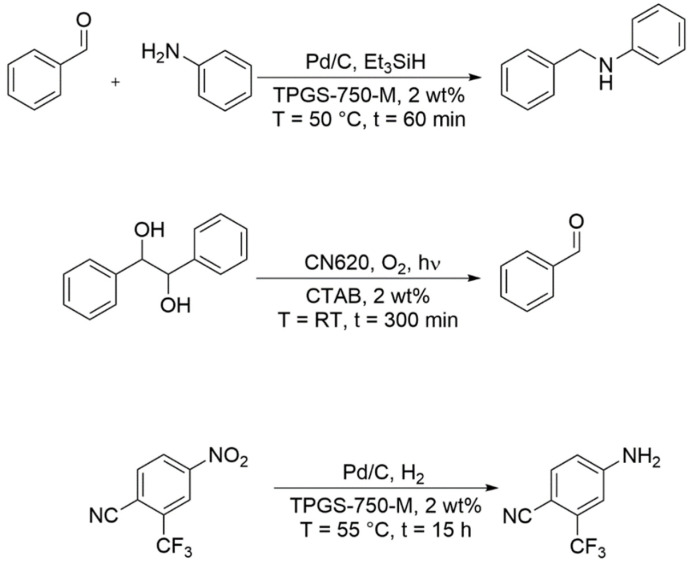
Examples of heterogeneous catalysts in micellar reaction medium.

Even if the characteristic properties of the surfactants allow very different separation concepts, the optimal adjustment of reaction and separation is certainly a challenge. Depending on the reaction system, a suitable separation method can certainly be identified, but in terms of green chemistry, it would be advantageous to combine the strengths of different systems to obtain the best possible performance. The strength of micellar solutions is the solubilization of hydrophobic substances in an aqueous environment. If this advantage is to be exploited in catalysis independent of the separation methods already mentioned, it is advisable to combine this with the advantage of heterogeneous catalysis and to use immobilized catalysts. Thus, the catalysts can be easily separated by standard methods after the reaction and only the reaction products remain in the micellar solution. Depending on the substance properties, the methods already mentioned may possibly be used for product fractionation, e.g., phenylacetic acid and 6-aminopenicillanic acid in the bio-catalytic hydrolysis of PenG, because they have very different partition coefficients [105]. Figure 23 shows selected examples for micellar catalysis with heterogeneous catalysts. Wernik et al. studied the reductive amination of benzaldehyde with aniline to *N*-benzylaniline in micellar solutions of different surfactants (2 wt%) using palladium nanoparticles supported onto carbon (Pd/C) as the catalyst. Using the designer surfactant TPGS-750-M, the best performance at 50 °C within a 60 min reaction time was obtained [112]. The reaction was studied first in batch mode and subsequently transferred into a continuous flow mode. For both cases, they identified reaction conditions where both conversion and selectivity were >99%. After the reaction, the suspended Pd/C catalyst was simply filtered off, washed with ethyl acetate and water, and dried before its reuse in subsequent reactions. There was almost no loss in the reaction performance within five subsequent runs. The photocatalytic oxidative C-C cleavage of (*R*,*R*)-hydrobenzoin to benzaldehyde was studied by Niu et al. with a nitrogen-vacancies-containing carbon nitride photocatalyst (CN620) under oxygen atmosphere and light irradiation in a micellar reaction medium [113]. From surfactant screening, an aqueous 2 wt% CTAB solution showed the best performance. Due to the low solubility of HB in water, conversion and selectivity were low at about 8% and 75%, respectively. However, in the micellar reaction medium conversion and selectivity were significantly increased to 94% and 90%, respectively, due to the better solubilization of (*R*,*R*)-hydrobenzoin. The product was extracted with ethyl acetate and the micellar solution containing the suspended photocatalyst could be reused for a minimum of ten runs showing a stable reaction performance. Although the authors did not show it, the system could be further optimized and photocatalysis could be performed with an immobilized CN620 photocatalyst under continuous flow conditions. The reduction of nitro groups, e.g., in 4-nitro-2-(trifluoromethyl)benzonitrile, with a Pd/C catalyst in a micellar solution was investigated by Li et al. [114]. Hydrogenations performed well in TPGS-750-M micellar solutions with much higher yields compared to water. The product was again separated with ethyl acetate and the catalyst-containing phase was reused showing the same yield for a second run. 

There are far more examples in the literature showing that it is beneficial to combine a micellar reaction medium for better solubilization of the hydrophobic reactants with a heterogeneous catalyst for easier separation. However, many examples use a dispersed heterogeneous catalyst (as a solid or as micellar-stabilized nanoparticles), which is recovered and reused after extraction of the product with mainly ethyl acetate as the solvent. With respect to continuous operation, further optimization is possible by immobilizing the catalyst and using the micellar solution only as the solvent for the reactants from which the product is separated. 

In terms of technical process, the added surfactant must fulfill different functions. On the one hand, it must support the solubilization of the reactants and the reaction. On the other hand, it must also support the separation process after the reaction. The examples shown in this section illustrate how the catalysts can be separated from the products in active form after the reactions. Independently, these methods can also be used to separate the surfactants themselves after the reactions. Depending on the surfactant class and surfactant concentration, the optimal separation process (filtration, extraction, or flotation) must be selected. The workup of the reaction mixture, e.g., to recover the surfactant, water, and other auxiliaries, needs deeper investigation in the future and might be challenging. Because of this, the attention of research groups is shifting towards the development of new surfactants, possibly from renewable resources, also investigating their biodegradability and reusability. Examples are reported in Section 2.10. 

### 2.10. Design for Degradation

Surfactants are frequently used in small amounts, avoiding the employment of large amounts of organic solvents that are non-biodegradable and persist long in the environment. Nevertheless, the chemical nature of surfactants poses many questions about their degradation and persistence in the environment. The large use of surfactants for detergency purposes over the years has led to a wide range of commercially available surfactants that are rather rapidly degraded. But due to their high affinity for water, the presence of these substances in water phases in the environment is unavoidable. Therefore, ideal surfactants should be composed of hydrophilic and hydrophobic parts that are easily separated, possibly leading to naturally occurring compounds that are rapidly degraded by microorganisms. The biodegradability and biocompatibility of synthetic surfactants has been recently reviewed [115]. A recent study investigated the issues posed by aqueous phases containing TPGS-750-M as an example of a versatile designer surfactant for micellar catalysis [116]. The specific surfactant cannot yet be classified as environmentally benign and possible actions involve its removal from water, for instance by extraction. This at least leads to a reduced CO_2_ footprint compared to incineration of the corresponding treated wastewater. This work emphasizes the importance of a complete investigation on the entire lifecycle of surfactants in a cradle to grave approach, considering not only the design, the function, and use in micellar catalysis, but also their final remains. It is advisable that emerging new designer surfactants are designed considering their final degradation to avoid persistence in the environment from the beginning. Just before submission of the present review, an interesting article appeared in the literature focusing on the carbon footprint of TPGS-750-M, under the viewpoint of possible employments in the pharmaceutical synthesis of API in water [117]. Overall, this designer surfactant is characterized by a carbon footprint that lies in the lower range of green solvents like methanol, methyl tert-butyl ether, heptane, toluene, and 2-methyl-tetrahydrofurane. This result further supports the development of micellar catalysis in industrial pharmaceutical production with the ultimate goal of large improvements in terms of reductions in fundamental parameters like the E factor and carbon footprint. To specifically tackle the biodegradability issues that characterize most designer surfactants based on the polyethylenglycol hydrophilic side chain, Lipshutz and collaborators designed a new surfactant combining the hydrophobic vitamin E with the hydrophilic polysarcosine unit characterized by a rapid N-methylglycine amide bond with minimization of downstream wastewater processing. The surfactant called Savie [118] can be synthesized in a one-pot approach with an overall 99% yield and one fourth of the waste generation compared to the well-established TPGS-750-M. Many practical advantages like a solid state at room temperature for improved shelf life, rapid dissolution in water, no presence of cloud point, and low solubility in organic solvents for improved aqueous phase recycling clearly speaks for the step forward achievable with this new surfactant. In terms of applications in micellar catalysis, the authors observed enhanced reaction rates and conversions for a multitude of reactions, avoidance of the use of organic co-solvents, and beneficial effects also in multistep chemoenzymatic catalysis.

### 2.11. Real-Time Analysis for Pollution Prevention

**Figure 24:**
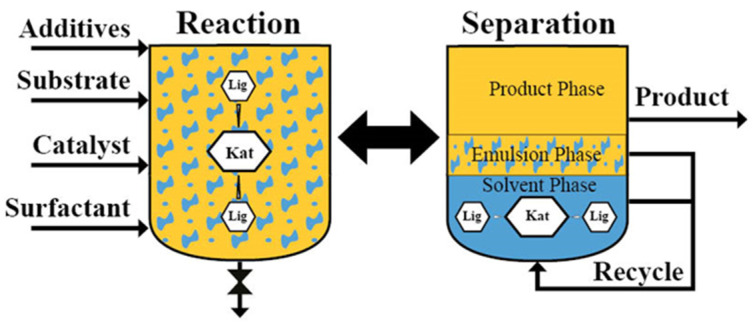
Process concept for homogeneous catalysis in microemulsions. Catalyst represents an aqueous catalyst solution consisting of water, catalyst precursor (Cat), and a ligand (Lig). Exploitation of phase behavior for product separation.

As already shown, chemical processes based on micellar systems include a variety of classes of new substances. Besides the catalytic systems consisting of enzymes [54,119], metal nanoparticles [53], or metal–organic complexes functionalized with ligands [120], applied amphiphiles or surface active compounds are the main functional elements. If an exemplary micellar system based on water as the main solvent is considered, such a system can be integrated as a production process in combining subsequent reaction and separation steps. Following Figure 24, three major goals can be derived regarding real-time analysis and pollution prevention: (i) Process analytical tools have to be available to track relevant compounds in the reaction process and subsequent downstream processes. This incorporates not only the product but also possible byproducts, which might accumulate within the process, waste, or product streams. Additionally, the catalytic system, amphiphiles, and additives have to be tracked to control the recycle operation, separation efficiency, or the possibly required replenishing of substances. (ii) These analytical tools need to furthermore enable process control and hence provide information within a suitable time of analysis (ideally real-time). (iii) The deployed analytical methods should adhere to the goals of green analytical chemistry and ideally generate information with a minimum of waste [121,122].

Regarding reaction monitoring, standard well-developed methods, such as gas chromatography and high pressure liquid chromatography are available. However, their online applicability is limited with increasing complexity in sample preparation and analysis time. Here, the applications of measurements based on spectroscopic methods are prone to provide in-situ online information. However, their application for micellar media is challenging because changes in the aggregation state and structural characteristics of micellar media alter the optical background of the reaction systems and hence influence signal intensities of specific analytes. This has been shown for the rhodium-catalyzed hydroformylation of 1-dodecene in a microemulsion system, using Marlipal^®^ 24/70 as a nonionic surfactant [123,124]. Although main reactants and byproducts are generally accessible via online in-situ Raman spectroscopy, reproducible quantification could not be achieved, as already small changes in the micellar system (change in temperature, concentration changes due to reaction progress, or recycling) perturbed the intensity signals. It is thus generally required to couple reaction tracking with tracking of the state of the emulsion (micellar or microemulsion system). Regarding this, several methods are available and have been already applied to track emulsions states. Amin et al. applied dynamic light scattering (DLS) for micro-rheologic characterization in combination with Raman spectroscopy to identify rheological properties and structural configurations of micellar systems with anionic and zwitterionic surfactants [125]. Early experiments also have already focused on Raman spectroscopy to determine and track transitions of phase states in binary and ternary microemulsion systems [126,127], as well as the influence of pressure and temperature on the shape of micelles [128]. 

### 2.12. Inherently Safer Chemistry for Accident Prevention

Intense activities in increasing the safety of chemical processes have been visible throughout the last decades. Following Kletz [129], the cornerstone in the prevention of hazards and risks is to initially strive for “friendly” plants, i.e., inherently safe plant design to meet E(nvironmental), H(ealth), and S(afety) criteria. Key aspects regarding applied materials and handling within plants are:

- S1 Intensification or minimization to reduce the amount of used or processed potentially hazardous materials
- S2 Substitution to reduce hazardous materials, intermediates, or utilities.
- S3 Attenuation by deploying hazardous materials under the least hazardous conditions (state, temperature, and pressure)

As discussed throughout this review, one of the key features of micellar catalysis and micellar systems in general is the incorporation of water as a solvent. In comparison to widely applied catalytic processes in organic solvent systems, micellar media hence inherently contribute to process safety and accident prevention regarding typical hazards, such as fires, explosions, or toxicity on release. S1 and S2 are consequently considered, as the amount of high-risk components is reduced or replaced by water, while maintaining catalytic activity [130]. As an example, the production of acyloxyketones and azidoketones, which are important intermediates with broad application as pharmaceutic products and specialties, is considered. Conventional production methods of these components are based on substitution reactions or oxidative coupling of carbonyl compounds in the presence of strong oxidants [131,132]. Most methods thus require the handling of explosive organic azides and peroxides [133], or toxic organic solvents [134]. This implies severe restrictions on the dimensions of operated plants, as well as adequate safety concepts. To overcome this hurdle, Handa et al. proposed the usage of a micellar system based on the amphiphile PS-750-M that mimics dipolar-aprotic solvents. This way, the oxyfunctionalization of styrenes in water under mild conditions is enabled in a one-pot solution based on *N*-bromo succinimide. The micelle structure and resulting local concentrations of reactants (dispersed organic phase and aqueous continuous phase) ensure a safe in-situ formation of radicals and their further reactions. Exploiting the micelles as spherical elements of scalable size, the upscaling of a respective process is attainable without invoking additional operational risks. Specifically, the micelles ensure that at least locally only a limited amount of reactant is available and enclosed by water as the continuous phase. Using the same approach, the oxyamination of styrenes is also reported.

Another beneficial contribution is realized in establishing rather mild process conditions (S3). In combination with larger quantities of water in the reaction system, the safe operation of processes with highly exothermic reactions becomes attainable. One example is the reduction of 4-nitrochlorobenzene, using Fe supported palladium nanoparticles, THF as an organic co-solvent, KBH_4_ as the hydrogen source, and the designer-made surfactant TPGS-750-M. The latter is applied as an aqueous solution, thus forming a microemulsion. Based on a 40 mmol scale and the reaction carried out in a 0.5 L reactor at 25 °C and 1 bar, a maximum adiabatic temperature increase of 37 °C is achieved. Despite the rather high heat of reaction of approx. −896 kJ/mol, a respective production process is proven to be safe as boiling conditions of the microemulsion, with water as the major component fraction, are not reached [135].

Although the chemical compositions and possible synthesis routes for using micellar systems, e.g., for catalysis, show tremendous potential for inherently safer processes, drawbacks regarding the physical properties of micellar systems or microemulsions are also visible. Considering a simplified ternary system of water, an oily substrate, and an amphiphile, a multitude of thermodynamic equilibrium states showing various (liquid) phase configurations are possible [136]. For nonionic amphiphiles with high (local) concentrations, lyotropic mesophases or liquid crystal phases can occur [137,138]. These are partly of high viscosity and lead to the blocking of pipes and pumps, resulting in hazards for process operation. This is especially relevant in downstream processes after the reaction step and for the recycling of amphiphiles if continuous production processes are considered. This challenge has already been addressed for the hydroformylation of 1-dodecene in a microemulsion system based on nonionic surfactants [76]. 

**Figure 25:**
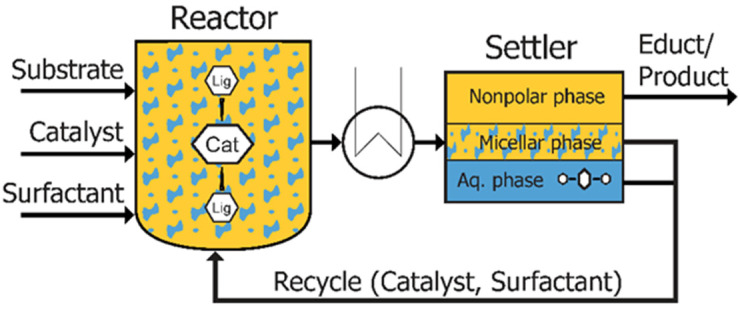
Process concept for homogeneously catalyzed hydroformylation of 1-dodecene in microemulsions. With precursor (Cat) and ligand (Lig) dissolved in an aqueous phase.

Using homogeneous catalysis based on the rhodium precursor acetylacetonato(dicarbonyl)rhodium(I) and SulfoXantPhos [75] as a ligand, the reaction leads to very high target product selectivity (>92%) under mild process conditions (95 °C, 15 bar) compared to standard industrial processes [139]. A mini-plant setup for continuous operation has been built up, consisting of a 1.5 L reactor, a subsequent settler, and three individual recycle streams (see Figure 25).

Via gravity-induced phase separation, the reaction mixture (microemulsion) is split into three liquid phases after the reaction—an oily product phase, a middle emulsion phase, and an aqueous catalyst-rich phase. All phases are recycled back to the reactor, while the oil stream is partially drawn off as a product stream. One of the crucial aspects in realizing stable long-term continuous operations with steady-state product yield was the determination and handling of operational challenges due to the phase separation of the microemulsion. This requires profound understanding of the phase separation behavior. In this case study, it is characterized by very small feasible operation regions, rather high sensitivity of the position of these operation regions regarding all concentrations, and the formation of viscous surfactant layers at the boundary of operation. The latter is prone to inhibit plant operation due to pump malfunction and accumulations of components. This further increases the risk for hazardous events, such as overheating equipment, bursting of pipes, or leakage. To overcome this hurdle, advanced strategies for process control, such as dynamic real-time optimization, have to be realized as an additional layer of protection [140] and for enabling stable process operation [141].

## 3. Conclusions and Perspectives

Green chemistry is a philosophy to make reactions and processes safer and more environmentally friendly. In principle, the twelve principles of green chemistry allow an improvement on very different levels and the implementation of the concept in full is often challenging. For each reaction to be carried out, it is necessary to consider in advance which measures are not only the most suitable but also readily implementable. This overview article focuses on micellar reaction media and shows how the twelve principles have been implemented so far or are attainable. Although there are certainly reactions that cannot be transferred to a micellar medium for various reasons, e.g., compatibility with the micellar medium or too low productivity, the number of positive examples is a confirmation for this medium. 

In recent years, the achievements of micellar catalysis not only in terms of improved yields, better selectivities, and simplified catalyst recycling but also process implementations are enabling a straightforward replacement of organic media and spreading the use of surfactants in water. This irreversible revolution in medium selection is further accelerated by the many advantages that micellar media provide when discussed through the lens of the twelve principles of green chemistry. The literature surveyed in this review demonstrates that all the green chemistry principles are largely represented by the use of micellar media, enabling in many cases multiple principles being met at the same time. It is not by chance that pharmaceutical industries, that are known for being highly sensitive to waste reduction and green metrics to improve the sustainability of their complex productions, are prone to implement micellar media for large-scale productions [142]. On a more academic level, future perspectives are possible in combining metal catalysis and surfactants as in metallosurfactants [143], as well as nanoparticles and surfactants [144]. Aiming at bridging the gap between metal and bio-catalysis, recent examples have further enlarged the field of application of micellar catalysis [145]. Last but not least, a major effort is now required for the development of a comprehensive toolbox of new biosurfactants completely based on renewable resources that ensures what is nowadays possible with synthetic surfactants. To demonstrate the rising impact of micellar catalysis in today’s catalysis, it is interesting to note that in a few months prior to finishing this contribution, several review articles dissecting many advantages of this catalytic method appeared in the literature [146,147,148]. Micellar catalysis has opened a doorway to the future, where water will be, like in nature, the solvent of choice for chemical reactions. Based on the multiple examples shown in this overview, which were chosen to represent and test the different principles of green chemistry, we can finally conclude: micellar catalysis is green chemistry! 

## Figures and Tables

**Table 1:** Selected EHS values from Capello et al. [68].

Solvent	EHS	Solvent	EHS	Solvent	EHS
Acetone	3.1	Acetonitrile	4.5	Cyclohexane	4.0
Dimethylether	3.9	Ethanol	2.6	Heptane	3.8
Methanol	2.7	Tetrahydrofurane	3.9	Water	0
